# Health, Costs, and Injection-Related Infections at a Hypothetical Overdose Prevention Center

**DOI:** 10.1001/jamanetworkopen.2025.55965

**Published:** 2026-01-28

**Authors:** Pranav Padmanabhan, Yjuliana Tin, Samantha K. Nall, Alia Al-Tayyib, Theodore Yoder, Kristina Yamkovoy, Kevin Fotso, Paul J. Christine, Cole Jurecka, Lisa Raville, Danielle M. Kline, Joshua A. Barocas

**Affiliations:** 1 Department of Medicine, Division of General Internal Medicine, University of Colorado School of Medicine, Aurora; 2 Department of Epidemiology, Colorado School of Public Health, Aurora; 3 Department of Health Systems, Management & Policy, Colorado School of Public Health, Aurora; 4 Denver Health and Hospital Authority, Denver, Colorado; 5 Office of Information Technology, University of Colorado School of Medicine, Aurora; 6 Harm Reduction Action Center, Denver, Colorado; 7 Department of Medicine, Division of Infectious Diseases, University of Colorado School of Medicine, Aurora

## Abstract

**Importance:**

While the impact of overdose prevention centers (OPCs) on fatal overdose, HIV, and hepatitis C is well-characterized, the long-term clinical and cost impact on injection-related infections is unknown, and empirical data from the US are limited.

**Objective:**

To estimate the 10-year impact of a hypothetical OPC in Denver, Colorado, on injection-related infections, hospitalizations, mortality, and associated costs from a payer perspective.

**Design, Setting, and Participants:**

This decision analytical modeling study used a calibrated and validated Monte Carlo microsimulation model of the natural history of injection drug use. Data included people who inject drugs (PWID) in Denver, Colorado, simulated over 10 years, from 2023 to 2032. Input data were largely collected from the 2022 National HIV Behavioral Surveillance survey and published research on currently operating OPCs.

**Exposure:**

Treatment service model for PWID; the status quo, in which 3 syringe service programs are operating, was compared with counterfactual scenarios in which a single OPC also operated and served between 10% and 70% of Denver’s population of PWID.

**Main Outcomes and Measures:**

Incidence of serious injection-related infections (SIRIs; infective endocarditis [IE] and skin and soft tissue infections [SSTIs]), hospitalizations, mortality, and associated costs from a health care payer perspective.

**Results:**

The modeled population of 9697 PWID had an input mean (SD) age of 41.3 (1.9) years and included 74.1% male participants. Over a 10-year period, the status quo resulted in approximately 15 400 SIRIs, 15 000 hospitalizations for SIRIs and overdose, and 2400 deaths among Denver’s population of PWID. Compared with the status quo scenario, a hypothetical OPC decreased SSTI incidence by up to 11.5% (95% credible interval [CrI], −16.8% to −6.4%), IE incidence by up to 22.0% (95% CrI, −27.6% to −6.1%), hospitalizations by up to 8.5% (95% CrI, −14.0% to −2.6%), and all-cause mortality by up to 5.8% (95% CrI, −12.4% to 4.9%), and saved between $7 million and $46 million from a payer perspective over 10 years, depending on the program reach. Findings were robust in sensitivity analyses.

**Conclusions and Relevance:**

In this simulation modeling study of the effect of a hypothetical OPC, implementing a single OPC in Denver was an effective intervention to decrease SIRIs, drug use-related hospitalizations, costs, and deaths among PWID.

## Key Points

**Question:**

What are the long-term clinical and cost impacts of overdose prevention centers on injection-related infections among people who inject drugs?

**Findings:**

In this study of a decision analytical model including US adults who injected drugs, an overdose prevention center in 1 urban center was projected to reduce skin and soft tissue infection incidence, infective endocarditis incidence, and hospitalizations for overdose and infections and save costs to payers over 10 years compared with the status quo of syringe service programs.

**Meaning:**

These results suggest that overdose prevention centers are cost-saving upstream interventions to mitigate injection-related infections and hospitalizations.

## Teaser

This decision analytical modeling study projects 10-year health and cost outcomes associated with a hypothetical overdose prevention center in a US city.

## Introduction

People who inject drugs (PWID) experience significant morbidity and mortality,^1^ largely attributable to overdose and infections like HIV, hepatitis C (HCV), and serious injection-related infections (SIRIs), including infective endocarditis (IE) and skin and soft tissue infections (SSTI).^2,3^ In 2017, there were approximately 20 000 hospitalizations for IE and 98 000 hospitalizations and emergency department visits for SSTI among PWID in the US.^4^ SIRI-related hospitalizations are increasing,^5^ with 1 study projecting nearly 260 000 deaths from IE among PWID in the US by 2030.^6^

Harm reduction strategies, including sterile syringe provision and naloxone distribution, have proven effective in mitigating risk for complications from drug use.^7,8^ Syringe service programs (SSPs) provide sterile injection equipment and naloxone, among other services, and are associated with a 46% to 54% reduction in odds of acquiring HIV^9^ and a 50% reduction in risk of acquiring HCV.^10^ Overdose prevention centers (OPCs) (sometimes called “supervised consumption sites”), are an emerging strategy in the US that expand the scope of existing harm reduction services by allowing individuals to legally bring, test, and use their own substances while being monitored by trained medical staff who can intervene in case of an adverse reaction.^11^ Like SSPs, OPCs also provide sterile injecting equipment and linkage to medical care, social services,^11^ and on-site wound care, which may abate abscesses and other SSTIs.^12^

OPC implementation in the US has been limited with 3 sanctioned OPCs currently operating, 2 in New York and 1 in Rhode Island. Nevertheless, results from these sites, other unsanctioned sites, and over 100 others in Canada, Australia, and Europe demonstrate that OPCs decrease emergency department visits, syringe sharing, and injection initiation, and increase uptake of treatment for substance use disorders.^13,14,15,16^ OPCs are cost-effective and decrease fatal overdose, HIV and HCV transmission, public drug use, and discarded needles.^17,18,19^

While the impact of OPCs on these outcomes is well characterized, how they affect long-term SIRI incidence and hospitalization is less well known.^20^ As SIRIs cause a large proportion of hospitalizations among PWID, mitigating the risk of SIRIs can alleviate the cost burden to health care systems. The long-term impact of OPCs on SIRIs may be important for state and local decision-makers considering policy tradeoffs and public health planning. Using a validated simulation model of injection drug use, we aimed to answer the question, “What are the long-term clinical impacts and cost-effectiveness of a hypothetical OPC on incidence of IE and SSTIs, fatal and nonfatal overdoses, hospitalizations, and mortality among a population of PWID in Denver, Colorado?” Simulation models can estimate costs and benefits of hypothetical public health interventions, and are particularly useful when proposed interventions are challenging to implement due to legal restrictions.

## Methods

We used the Consolidated Health Economic Evaluation Reporting Standards (CHEERS) to guide the writing of this manuscript. This study was approved by the Colorado Multiple Institutional Review Board as an exempt study due to use of deidentified data.

### Model Description

We used the Reducing Infections Related to Drug Use Cost Effectiveness (ReDUCE) model, an established Monte Carlo microsimulation model of the natural history of injection drug use, to estimate the long-term clinical and cost outcomes associated with opening a hypothetical OPC. The main outcomes in the model include SIRIs (IE and SSTIs), fatal and nonfatal overdoses, hospitalizations, and all-cause mortality among a cohort of PWID over a 10-year period (2023-2032).^21^ Denver was chosen as the study city due to data availability and policy relevance, as Denver City Council has approved a pilot OPC but the state legislature has requested more locally tailored data.^22^

In the model, simulated cohorts are assigned demographics and behavioral characteristics reflecting the study population. Individuals encounter weekly individual-dependent probabilities of SIRIs, overdose, hospitalization, outpatient care, behavioral transitions, and death. Full model details are in the eAppendix in Supplement 1.

### Model Analyses

**Figure 1.**
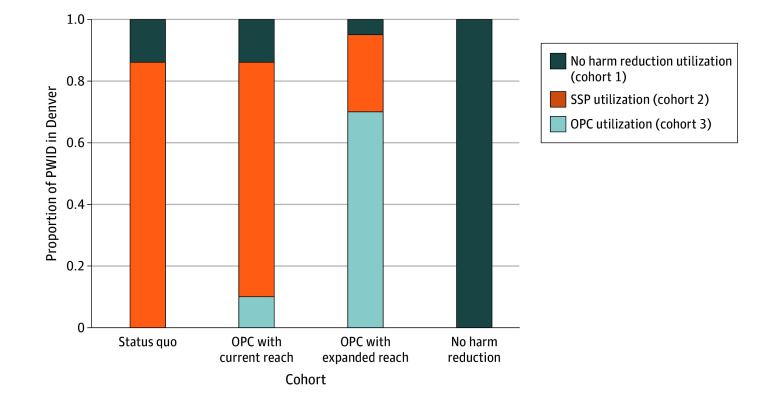
Harm Reduction Service Utilization Among PWID Under Various Scenarios Involving OPC Implementation OPC indicates overdose prevention center; SSP, syringe service program; PWID, people who inject drugs.

We compared 3 scenarios: (1) *status quo*, in which SSPs are currently operating without an OPC; (2) *OPC with current reach*, in which an OPC is implemented and used exclusively by a fraction of individuals who are already using an SSP (ie, most individuals remain using an SSP exclusively); (3) *OPC with expanded reach*, in which an OPC is implemented resulting in increased overall utilization of harm reduction services to 95% of Denver’s population of PWID (ie, some individuals not currently using SSPs will use an OPC) (Figure 1).

We generated composite outputs for each scenario by calculating population-weighted averages of 3 simulated cohorts: (1) PWID who did not use either an SSP or OPC; (2) PWID who used an SSP only; and (3) PWID who used an OPC.

We assumed that OPCs offer all services provided at SSPs, including naloxone and sterile injection equipment and linkage to medical care and social services.^23^ Unless suggested by treatment literature, we assumed that demographics and behaviors of individuals using OPCs were equivalent to those using SSPs. Compared with SSP users, OPC users received a greater probability of skin cleaning and lower probabilities of needle sharing and overdose. Input parameters by cohort are shown in Table 1, and full parameterization processes are described in eTable 1 in Supplement 1. We assumed that individuals do not transition between cohorts within the simulation period. We did not assume that individuals use an OPC for every injection event; therefore, individuals in this cohort may still fatally overdose despite no record of fatal overdoses at OPCs. Similarly, probabilities of infection among those using OPCs reflect that some injections occur outside of OPCs.

**Table 1.** Selected Model Inputs for Analysis of Clinical Impacts and Cost-Effectiveness of OPC Implementation

Parameter	PWID, No. (%) (N = 9697)^a^		
	No harm reduction	SSPs only	OPCs
Age, mean (SD), y	42.3 (2.5)	41.1 (1.8)	41.1 (1.8)
Sex			
Male	6613 (68.2)	7282 (75.1)	7282 (75.1)
Female	3084 (31.8)	2415 (24.9)	2415 (24.9)
Injection behaviors			
High-frequency injection	4645 (47.9)	7399 (76.3)	7399 (76.3)
Low-frequency injection	1629 (16.8)	1270 (13.1)	1270 (13.1)
No current injection	3423 (35.3)	1028 (10.6)	1028 (10.6)
Unsterile injection	2812 (29.0)	1202 (12.4)	553 (5.7)
Sharing needles	1493 (15.4)	1348 (13.9)	262 (2.7)
**Sequelae of drug use**			
Probability of overdose^b^			
High-frequency injection, estimated events/wk	0.01300	0.01300	0.01275
Low-frequency injection, estimated events/wk	0.0050	0.0050	0.0049
Probability of fatal overdose, conditional on overdose, estimated events/wk^b^	0.08	0.056	0
**Outpatient services**			
Weekly probability of MOUD initiation, estimated events/wk^b^	0.0121	0.0171	0.0171
Healthcare costs, 2022 US$^b^			
SSTI hospitalization	20 227	20 227	20 227
IE hospitalization	24 582	24 582	24 582
Overdose hospitalization	16 175	16 175	16 175
Addiction consultation	256	256	256
Infectious disease consultation	922	922	922
OPAT	179	179	179
MOUD	50	50	50
Antibiotics	279	279	279
Addiction care with MOUD	135	135	135
Addiction care without MOUD	92	92	92

Abbreviations: IE, infective endocarditis; MOUD, medications for opioid use disorder; OPAT, outpatient parenteral antibiotic therapy; OPC, overdose prevention center; PWID, people who inject drugs; SSP, syringe service program; SSTI, skin and soft tissue infections.

^a^ Population parameters for cohorts 1 and 2 were taken directly from National HIV Behavioral Surveillance data.

^b^ All probabilities and costs represent a weekly cycle.

In the status quo scenario, the proportion of PWID who used OPCs was 0%, as OPCs are not currently operating. In the OPC with current reach scenario, the population of OPC users was drawn from the population of SSP users in the status quo, and the proportion of PWID who do not use SSPs was unchanged. In the OPC with expanded reach scenario, the proportion of OPC users was drawn from those who did and did not use SSPs. The proportion of OPC users in this scenario was 70%, an estimate of individuals willing to use OPCs drawn from multicity surveys.^24^

### Statistical Analysis

We estimated 10-year incidence of IE and SSTIs; fatal and nonfatal overdose; hospitalizations for IE, SSTIs, and/or overdose; mortality; and costs (in 2022 US$) from population-weighted averages of the model output, which are reported with 95% credible intervals (95% CrI). We used standard methods to calculate the incremental cost-effectiveness ratios of each treatment strategy.^25^ We projected 10-year medical costs (including hospital and outpatient medical care) assuming a payer system perspective and applied a 3% discount rate to costs and life-years.^25^ The majority of payers for drug use-related hospitalizations are Medicaid or self-pay.^26^ Model output was analyzed using Excel (Microsoft Corp) and R version 4.2.3 (R Project for Statistical Computing).

#### Model Parameterization and Cohort Design

We calculated model parameters largely using the 2022 National HIV Behavioral Surveillance (NHBS) survey of PWID in Denver, Colorado; treatment literature from sanctioned OPCs in New York, Canada, and France and unsanctioned OPCs elsewhere in the US; and other national cohort studies (eTable 1 in Supplement 1). We initialized the model to reflect the age and sex of PWID in Denver. To limit Monte Carlo variance, we modeled a large cohort size of 1 000 000 individuals and scaled output proportionally to an estimated population of 9697 PWID in Denver (derivation in eAppendix in Supplement 1). As reported by NHBS, 86% of PWID use an SSP in the status quo scenario, while 14% do not utilize formal harm reduction services.

#### Outcomes and Measures

We used published research, including a meta-analysis, to estimate the rates of SIRIs and fatal and nonfatal overdose.^2,4,27,28^ Sequelae were considered severe enough to require hospitalization to be treated. For outpatient services, we derived probabilities of linkage to addiction care, with or without MOUD, after hospital discharge and via outpatient connection to care from cohort studies.^29,30,31^

#### Costs

Individuals accrue weekly costs related to disease status and hospital and outpatient service utilization. The costs of OUD care vary by injection behavior profile, with additional costs accrued by those with higher-frequency use. Initial and weekly costs of MOUD and age- and sex-stratified costs of health care services not attributable to opioid use or sequelae were sourced from the Medical Expenditure Panel Survey and the Centers for Medicare & Medicaid Services Physician Fee Schedule.^32,33^

#### Calibration

We calibrated the model to national and local outcomes, including SIRI hospitalization incidence and mortality, fatal and nonfatal overdose incidence, and all-cause mortality.^1,28,34^ We assessed external validity by comparing the 1-year model-predicted fatal overdose incidence among Denver’s population of PWID in 2022 to the number of fatal overdoses recorded by the Colorado Department of Public Health and Environment. Calibration and validation procedures are described in eAppendix in Supplement 1.

#### Sensitivity Analyses

We conducted deterministic and probabilistic sensitivity analyses (DSAs and PSAs) to discern robustness of model estimates. DSAs included best case and worst case scenarios utilizing 95% CIs of odds ratios associated with differences in injection behaviors between OPC and non-OPC users.^16,35^ The best case scenario assumed maximum odds of skin cleaning and minimum odds of needle sharing associated with OPCs, and vice-versa in the worst case. We also varied reductions in overdoses associated with OPCs. PSAs involved 1000 simulations of 1000 individuals each per cohort, maintaining a total simulated population of 1 000 000 individuals, in which uncertain input parameter values were sampled from distributions to form unique sets for each simulation. We report PSA results as 95% CrIs of model estimates.

#### Alternative Scenarios

Services offered at OPCs and implementation processes vary. We conducted scenario analyses in which OPCs followed a community health center-embedded (CHC) rather than the more common harm reduction–embedded model. Harm reduction models typically have higher uptake of harm reduction practices, but CHC models have higher linkage to MOUD.^23^

We also considered a scenario in which harm reduction services are removed (ie, no harm reduction). We conducted this scenario as syringe distribution bans have been implemented elsewhere in Colorado,^36^ as well as West Virginia^37^ and California.^38^

## Results

In the status quo, the study population of 9697 PWID in Denver, an aggregate of simulated cohorts, had a mean (SD) age of 41.3 (1.9) years (Table 1). The model population included 7185 male participants (74.1%); 7021 (72.4%) injected at high frequency (at least once a day), 1319 (13.6%) injected at low frequency, and 1357 (14.0%) were not currently injecting; 1425 (14.7%) did not regularly clean their skin prior to injecting, and 1367 (14.1%) regularly shared needles.

### Clinical Outcomes

**Figure 2.**
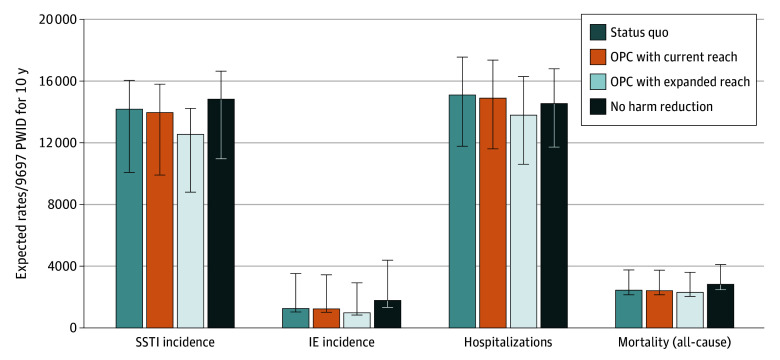
Ten-Year Clinical Outcomes (2022-2032) Among PWID by Scenario IE indicates infective endocarditis; OPC, overdose prevention center; PWID, people who inject drugs; SSTI, skin and soft tissue infection. Error bars represent 95% credible intervals (CrIs) for each clinical outcome based on outputs of 1000 independent runs, each drawing unique sets of uncertain parameters (eg, the weekly probability of an individual with an SSTI linking to inpatient care) from given distributions. Error bars between scenarios may overlap, even when the 95% CrI for the change in an outcome between scenarios (reported in the text) does not include 0%. This is because the reported outcome of percentage change in a given clinical outcome between 2 scenarios was calculated assuming that the probabilistically drawn set of uncertain input parameters is the same in both scenarios being compared (ie, scenarios are not independent of one another).

In the status quo, the model estimated 1241 IE cases (95% CrI, 1017-3507), 14 184 SSTI cases (95% CrI, 10 081-16 058), 1106 fatal overdoses (95% CrI, 759-2077), and 16 778 nonfatal overdoses (95% CrI, 12 458-31 793) over a 10-year period (Figure 2; Table 2; eTable 6 in Supplement 1). The OPC with current reach scenario generated a decrease in IE incidence of −2.5% (95% CrI, −3.6% to −0.3), a decrease in SSTI incidence of −1.6% (95% CrI, −2.4% to −0.8%), an increase in fatal overdoses of 0.5% (95% CrI, −1.2% to 3.4%), and an increase in nonfatal overdoses of 0.5% (95% CrI, −0.3 to 2.2), while the OPC with expanded reach scenario generated a decrease in IE incidence of −22.0% (95% CrI, −27.6% to −6.1%), a decrease in SSTI incidence of −11.5% (95% CrI, −16.8% to −6.4%), an increase in fatal overdoses of 3.1% (95% CrI, −8.1% to 22.8%), and an increase in nonfatal overdoses of 6.2% (95% CrI, 0.8% to 17.6%).

**Table 2.** Changes in Clinical Outcomes by Scenario

Strategy	Estimate, % (95% CrI)			
	Change in SSTI incidence^a^	Change in IE incidence^a^	Change in SSTI, IE, and overdose hospitalization rate^a^	Change in all-cause mortality rate^a^
OPC with current reach	−1.6 (−2.4 to −0.8)	−2.5 (−3.6 to −0.3)	−1.3 (−2.0 to −0.4)	−0.6 (−1.6 to 0.9)
OPC with expanded reach	−11.5 (−16.8 to −6.4)	−22.0 (−27.6 to −6.1)	−8.5 (−14.0 to −2.6)	−5.8 (−12.4 to 4.9)
OPC with current reach (community health center model)	0.8 (−0.4 to 2.7)	−3.3 (−3.8 to 0.7)	1.5 (0.2 to 3.5)	−2.5 (−2.9 to −0.6)
OPC with expanded reach (community health center model)	5.3 (−3.3 to 17.9)	−27.5 (−29.6 to 2.1)	10.6 (1.8 to 24.2)	−19.5 (−21.2 to −5.6)
No harm reduction	4.6 (−2.4 to 15.0)	41.7 (7.4 to 52.2)	−3.7 (−12.1 to 5.8)	15.9 (−0.2 to 25.3)

Abbreviations: IE, infective endocarditis; OPC, overdose prevention center; SSTI, skin and soft tissue infections.

^a^ Percentage differences are in comparison with the status quo.

The status quo projected 15 049 hospitalizations for SIRIs and/or overdose (95% CrI, 11 780-17 565) over 10 years. OPC with current reach and OPC with expanded reach generated decreases in hospitalizations of −1.3% (95% CrI, −3.6% to −0.3%) and −8.5% (95% CrI, −14.0% to −2.6%) over 10 years, respectively. The status quo generated 2421 deaths over 10 years (95% CrI, 2137-3750). Mortality decreased by −0.6% (95% CrI, −1.6% to 0.9%) and −5.8% (95% CrI, −12.4% to 4.9%) in OPC with current reach and OPC with expanded reach respectively.

### Costs

**Figure 3.**
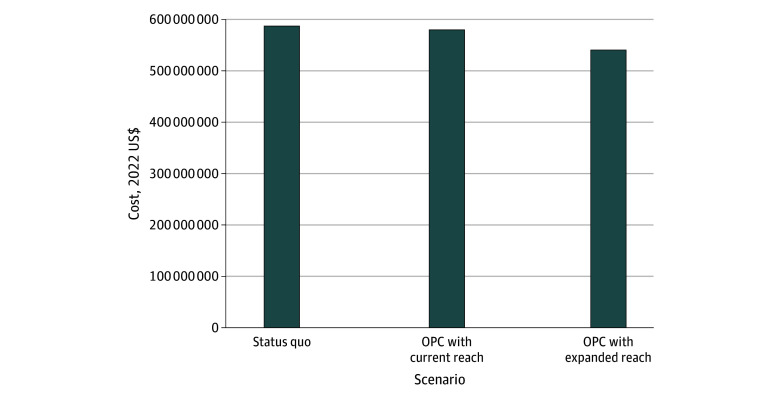
Total Costs to Health Care Payers Over 10 Years (2023-2032) Under Various Scenarios Involving OPC Implementation OPC indicates overdose prevention center.

Under the status quo, the average discounted per-person costs to the payer over 10 years was $387 700 (95% CrI, $358 500-$401 200). The total cost to payers over 10 years was approximately $587 million. Costs were largely attributable to hospitalizations for SIRIs and/or overdose ($488 million [83.1%]). OPCs were associated with decreased average discounted costs: OPC with current reach would result in average per-person costs of $387 400 (95% CrI, $358 300-$400 700) while OPC with expanded reach would result in average per-person costs of $387 400 (95% CrI, $358 500-$398 900). Total costs decreased by $7 million to $46 million over 10 years (Figure 3). Costs attributable to hospitalization did not change substantively between scenarios, ranging from 80.9% under the OPC with expanded reach scenario to 82.9% under OPC with current reach.

### Cost Effectiveness

Both the OPC with current reach and OPC with expanded reach scenarios dominated the status quo and are cost-saving. Compared with the status quo, the OPC with current reach scenario saved $300 in discounted costs per person over 10 years, and increased average discounted life span by 0.007 years. The OPC with expanded reach scenario projected increased life expectancy compared with OPC with current reach, saving $300 in discounted costs per person and increasing average discounted life span by 0.064 years compared with the status quo, making it the most cost-effective strategy (Figure 3).

### Sensitivity Analyses

We performed 1-way deterministic sensitivity analyses, adjusting uncertain parameters that may influence infection incidence and hospitalization. We found that needle sharing had the greatest effect on infection incidence (eTables 3 and 4 in Supplement 1).

### Alternative Scenarios

We evaluated scenarios in which OPCs followed a community health center (CHC) model, which emphasizes MOUD rather than sterile injection practices. In contrast to OPCs following a harm reduction model, OPCs following a CHC model generated increased SSTIs and more greatly reduced IE (both including no change within the 95% CrIs) increased hospitalizations, and more greatly reduced mortality compared with the status quo (Table 2). Under no harm reduction approaches, projected SSTIs, IE, and mortality increased while hospitalization decreased compared with the status quo, although most estimates had 95% CrIs inclusive of no change (Table 2).

## Discussion

In this modeling study, we examined the long-term impact of OPC implementation on SIRIs, hospitalizations, mortality, and costs among PWID in Denver, Colorado. OPCs resulted in fewer infections and deaths and reduced costs to payers compared with the status quo of only SSPs operating. IE incidence and hospitalization were more greatly reduced compared with SSTI incidence and hospitalization. Untreated SSTIs can progress to more complicated IE, so the benefit of SSTI prevention is likely multiplied downstream.

While SIRI incidence, hospitalizations, and deaths decreased, there was a nonintuitive finding of slightly increased fatal and nonfatal overdoses over 10 years in scenarios with OPC implementation. However, 95% CrIs for changes in fatal and nonfatal overdoses contained no change, except for nonfatal overdoses under OPC with expanded reach. A null association between OPC use and overdose aligns with findings from existing OPCs.^39,40^ In both modeled OPC scenarios, increases in nonfatal overdoses were larger than fatal overdoses. Potential increases could be attributed to longer life spans during which individuals can accumulate overdoses. Also, as SIRI incidence decreases, competing risks for death from infections are lessened. As OPCs roll out across the US, program evaluators should concurrently assess SIRI outcomes alongside overdoses to broadly capture impacts on multiple competing risks.

Our analysis adds to the growing body of US-based studies on OPCs by demonstrating that OPCs, alongside SSPs, can mitigate the profound burden of SIRIs and associated costs on individuals and the health care system. SIRIs cost 1 safety net hospital in Miami $11.4 million over 1 year in 2013-2014,^41^ and over $150 million to all Oregon hospitals in 2018.^42^ In our analysis, OPCs following a harm reduction model resulted in modest decreases in SIRIs, hospitalizations, deaths, and costs compared with the status quo. As these hospitalizations are largely self-pay, hospitals absorb a significant amount of costs. Therefore, preventing hospitalizations among this population should be a priority for administrators. Implementing a single OPC in Denver could save payers approximately $7 million to $46 million in undiscounted health care costs over a 10-year period.

Our results align with findings from OPCs in Toronto and Montreal showing modest decreases in infection and hospitalization incidence.^43,44,45^ While OPCs may decrease sequelae and health care costs, PWID often still use substances outside an OPC, due to factors including inaccessibility, limited hours of operation, distrust of medicalized harm reduction services, police presence around OPCs, privacy concerns, and personal preference.^44,46,47,48^ OPCs are a crucial component of the health care continuum for PWID, but social and logistical limitations of OPCs are unlikely to be wholly removed within sociopolitical environments which still criminalize and stigmatize substance use beyond their walls.

We explored variability around OPC service models in sensitivity analyses. Under community health center models, hospitalizations increased compared with the status quo, opposite to a harm reduction model. SSTIs also increased while IE decreased. OPCs following a CHC model may emphasize early medical care, which may increase health care utilization and prevent progression to more serious infections and death. Mortality decreased further under the CHC model compared with the harm reduction model. If SSPs were abolished, hospitalizations decreased but SSTI, IE, and mortality increased, suggesting that without access to harm reduction services, more individuals acquire severe infection and die before receiving medical care.

### Limitations

Our analysis includes several limitations. First, there was a lack of local data to fully inform our model. We addressed this by calibrating to Denver-specific data on overdose and MOUD initiation. Our model assumes that people who utilize an OPC are injecting drugs; we did not model those who are consuming via other routes. We could not account for all potential benefits of an OPC, including decreased stigma associated with using substances in hygienic and private locations and the benefits of OPCs as community spaces.^49,50^ While increased treatment access and retention associated with OPC use could be modeled, we did not model the downstream effects of OPCs as access points for housing and other social services. Therefore, estimates of intervention effects are likely conservative. Future work may explore the impact of OPCs on stigma and socioeconomic outcomes including access to housing, and the differential impact of OPCs across subgroups of PWID and people who use substances through noninjection routes of administration.

## Conclusions

The findings of this simulation study of the effect of a hypothetical OPC in Denver suggested that an OPC may improve health outcomes and save payers money in the long-term. Benefits were amplified by increasing harm reduction service capacity and removing barriers to utilization.

## Notes

Supplement 1. eAppendix. REDUCE model overview eTable 1. Input parameter values across simulated cohorts eTable 2. Calibration targets and modeled output eTable 3. Changes in outcomes in DSAs under OPC with current reach scenario eTable 4. Changes in outcomes in DSAs under OPC with expanded reach scenario eTable 5. Selected model inputs for analysis of clinical impacts and cost-effectiveness of OPC-CHC implementation eTable 6. Selected clinical outcomes by scenario eReferences.

Supplement 2. Data Sharing Statement
