# Supplementary material for: Health, Costs, and Injection-Related Infections at a Hypothetical Overdose Prevention Center
Source: JAMA Netw Open. 2026 Jan 28;9(1):e2555965. doi: 10.1001/jamanetworkopen.2025.55965 (PMC12853211; doi:10.1001/jamanetworkopen.2025.55965)
Supplement: Supplement 1. — eAppendix. REDUCE model overview eTable 1. Input parameter values across simulated cohorts eTable 2. Calibration targets and modeled output eTable 3. Changes in outcomes in DSAs under OPC with current reach scenario eTable 4. Changes in outcomes in DSAs under OPC with expanded reach scenario eTable 5. Selected model inputs for analysis of clinical impacts and cost-effectiveness of OPC-CHC implementation eTable 6. Selected clinical outcomes by scenario eReferences. [file jamanetwopen-e2555965-s001.pdf]

Supplemental Online Content

Padmanabhan P, Tin Y, Nall SK, et al. Health, costs, and injection-related infections at a hypothetical overdose prevention center. *JAMA Netw Open.* 2026;9(1):e2555965. doi:10.1001/jamanetworkopen.2025.55965

eAppendix. REDUCE model overview

eTable 1. Input parameter values across simulated cohorts

eTable 2. Calibration targets and modeled output

eTable 3. Changes in outcomes in DSAs under OPC with current reach scenario

eTable 4. Changes in outcomes in DSAs under OPC with expanded reach scenario

eTable 5. Selected model inputs for analysis of clinical impacts and cost-effectiveness of OPC-CHC implementation

eTable 6. Selected clinical outcomes by scenario

eReferences.

This supplemental material has been provided by the authors to give readers additional information about their work.

© 2025 Padmanabhan P et al. *JAMA Network Open*.

eAppendix. REDUCE Model Overview

Model description

The Reducing Infections Related to Drug Use Cost Effectiveness (ReDUCE) Model is an individual-level stochastic microsimulation model that simulates the natural history of injection opioid use in the U.S. for the purpose of forecasting injection-related bacterial infections. The model uses injection frequency and injection practices as the framework in order to estimate outcomes of interest including incidence of infections and deaths on a weekly basi. This simulation modeling framework can help demonstrate how interventions might yield long-term benefits and to measure the potential magnitude of those benefits for people who inject drugs.

The analyses reported in the main manuscript use the Reducing Infections Related to Drug Use Cost Effectiveness (REDUCE) Model of acquisition and treatment for bacterial infections and overdose associated with injection drug use. This technical appendix provides details on key features of the model and modeling approach used for this analysis. We constructed the model and performed analyses using C++ and R (3.2.2). The model has been previously described in the peer-reviewed literature.[1,2] The model is available for review upon discussion with the authors and as resources are available. We did not use every component of the model for the current analysis. In addition, we provide figures and several tables detailing input parameter values, calibration, validation, and additional results cited in the manuscript.

Overview

The model is designed as a number of modules through which simulated individuals pass. Briefly, a cohort module helps to "create" the population of interest. Next, individuals created during cohort generation enter the "sequelae of drug use (SDU)" module, which is where they encounter probabilities of fatal or nonfatal overdose, infective endocarditis, or skin and soft tissue infections. From the SDU module, individuals enter back into the simulation or link to the "inpatient" module. In the "inpatient" module, individuals are hospitalized for their SDU. There are a variety of interventions (beyond standard hospital treatment) that individuals may encounter if those services are turned "on" by the user. Following the inpatient module, individuals have a probability of linking to outpatient care in the "outpatient" module. Linkage to outpatient care may vary based on the type of services an individual encountered in the hospital and/or the type of SDU they have (overdose vs infection). They may unlink from the outpatient module or never enter it (based on probabilities). The "behavioral transitions" module is when individuals have the probability of moving between injection frequency drug use states (high frequency, low frequency, or no current drug use), between sterile injection practice states (skin cleaning or no skin cleaning), and sharing/reusing needles. After the "behavioral transitions" module, individuals move to the "mortality, cost, and quality of life" module. At this point, the model begins again in cycle n+1.

© 2025 Padmanabhan P et al. *JAMA Network Open*.

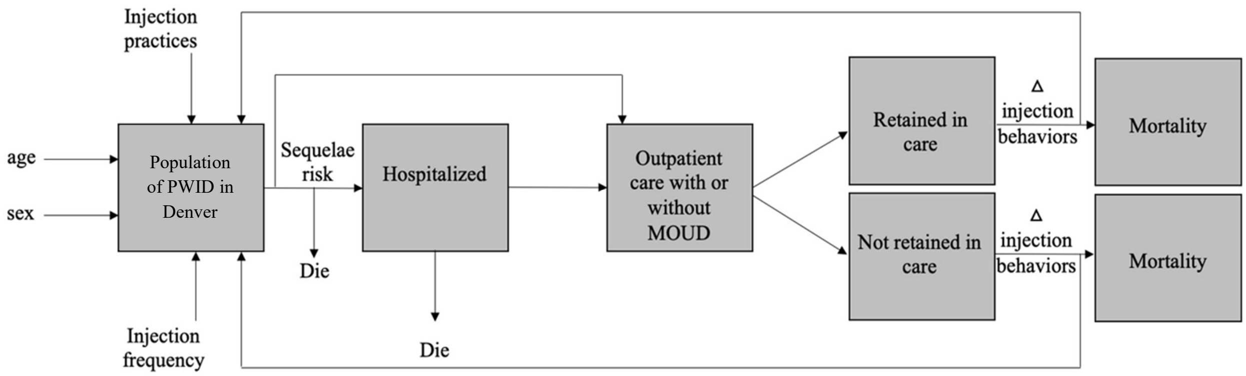

Fig. S1: REDUCE Model Schematic

Module 1: Cohort initiation. When the model is initiated, a cohort of individuals is generated using 6 parameters:

(1) ever injection drug use status (ever/never)
(2) age (0-99). If we only want a certain age range, leave the unwanted ages as 0.
(3) sex (M/F)
(4) injection frequency (high/low/no current/never)
(5) reusing/sharing equipment (yes/no/never).
(6) sterile injection practice (cleaning/no cleaning/never)

From these parameters, the cohort developed is a distribution of people who have ever or never injected drugs and those who are ever are stratified by injection frequency and injection practices. The model is structured such that first the user specifies the proportion of the population that has ever injected drugs. Following that, there are two methods by which the model can draw age and sex. The first is by using age/sex tables and the second is by directly specifying age and sex distribution parameters in a cfg file. For the former, there is an "ever_distribution.csv" table and a "never_distribution.csv" table.

Next, among those who are ever drug users, the probability of injection frequency is drawn from an age/sex stratified sql table—high, low, and no current injection drug use. For this, all three probabilities of an age/sex group should equal to 1 and the model draws from this set of probabilities. For the probabilistic sensitivity analysis component of the model, since all probabilities are drawn from distributions, the user specifies two of the input distributions and the 3rd input is the difference. The model does not allow for the added probability to be greater than 1. Finally, all persons who are ever drug users, are assigned an initial status of being a skin cleaner and a needle sharer which does not depend on age and gender. While these are the initial attributes, all individuals have the possibility of "picking up" additional attributes as they move through the model. All never drug users are assigned "never" injection frequency, skin cleaning and needle sharing status.

Assumptions built into the model for the initial cohort:

© 2025 Padmanabhan P et al. *JAMA Network Open*.

1) no one starts on treatment for opioid use disorder
2) no one starts out with a history of overdose
3) no one starts with a history of infection
4) no one begins in care or in the hospital setting.

Module 2. Sequelae of Drug Use. Once the cohort is initialized and each individual has been assigned an initial drug use status, age, sex, and injection frequency and practices, individuals enter the sequelae of drug use (SDU) module. Broadly, the SDU in this model include infective endocarditis (IE), skin and soft tissue infections (SSTIs), and overdose (OD). When they first enter, the model checks their ever/never status. If they are "never," then they return to the simulation. Therefore, only "ever" drug users can progress through this module. The model then checks their injection frequency. If they are "no current," then they should return to the simulation. Therefore, only "low frequency" and "high frequency" injectors will progress through this module. Additionally, if the individual is currently in inpatient care, they will return to the simulation. If a person is currently on antibiotics, they will progress through the SDU module but they cannot acquire a new infection (SSTI or IE).

At this point, remaining individuals are subject to probabilities for acquiring an SDU. On the first cycle of this model, no one has a history of SDU, but should have the possibility of acquiring one or multiple through their life. History of SDU is tracked as it has implications for future SDU. One assumption of the model is that in each cycle, an individual can have more than one SDU, but only acquires one infection at a time. Additionally, individuals may have concurrent SDU (meaning that they may acquire IE in cycle 1 and then SSTI in cycle 2, if they have not been hospitalized for their existing SDU. Another assumption of the model is that SDUs can only be acquired while not "inpatient" or on antibiotics (next module).

Individuals who are eligible for an SDU, then progress through a number of probabilities of acquiring an SDU. All SDUs are stratified by injection frequency (high and low) and the infectious SDU are also stratified by injection practices (skin cleaning, needle sharing). SDU probabilities are not stratified by age and sex. Since overdose is generally more common than infectious SDUs, the model is structured such that an individual first encounters a combined probability of overdose (fatal + nonfatal), stratified by injection frequency. If an individual has a current infection their overdose rate is multiplied by the current infection multiplier. A proportion of overdoses are fatal and a proportion are nonfatal. If a person draws an overdose, the model checks whether they are within their OEND effective cycles and dependent on that, fatal overdose is drawn. One aspect of the model is that at this point, if a person draws a fatal overdose then they are flagged as "dead, fatal overdose." They can continue to proceed through the rest of the modules but cannot acquire any further attributes (e.g., they cannot get another infection, be hospitalized, start MOUDs, change their behaviors). These individuals should, however, accrue the full costs of the cycle (based on background costs, costs of fatal overdose, and costs of any other SDUs that are untreated) and utilities (based on age, sex, and other current

health states at the end of the cycle). For those that have a nonfatal overdose or do not have an overdose, they then face a combined probability of infectious SDU (IE + SSTI), stratified by injection frequency, skin cleaning and needle sharing attributes. A proportion of that probability is IE and the other is SSTI. The model is structured to account for a history of SDU (treated in hospital, resolved because it was a nonfatal OD) and for existing SDUs. An existing SDU is anything that an individual has during the current cycle. From a clinical perspective, this represents an "untreated" infection (e.g., someone has not gone to the hospital for their endocarditis or someone is currently on outpatient antibiotics but not cured) or a current nonfatal overdose. Once treatment is complete or the SDU resolves (as is the case with nonfatal overdose which resolves in 1 cycle), then the person is flagged with a history of the corresponding SDU.

An existing SDU causes a change in the likelihood of another SDU. In the model, there is a single multiplier for one or more existing SDUs that is applied to both the probability of OD and the probability of infectious SDU. This multiplier exists until the individual is treated for the SDU. For those who have a nonfatal overdose, the existing SDU multiplier will be applied to the probability of infection in the same cycle only since an existing nonfatal OD (that does not link to inpatient), only lasts one cycle. Additionally, a history of SDUs changes the probability of future SDUs. Multipliers are only applied to the SDU for which there is a history (e.g., OD history changes the probability of recurrent OD; any infection history changes the probability of future infection [any infection, not just the one that occurred]). For OD, there are 4 multipliers (e.g., 1 past nonfatal OD, 2-3 past nonfatal OD, 4-7 past nonfatal OD, and 8+ past nonfatal ODs). For history of treated infections, there is only one multiplier (1+ past treated infections). For instance, in cycle 1, an individual gets IE but does not go to the hospital/receive treatment and does not die in cycle 1. By cycle 2, having IE makes that individual have a greater probability of OD or SSTI. For this model, individuals will not be able to acquire the same SDU in that next cycle. From the previous example, the individual with IE will only be able to acquire OD or SSTI, not IE in cycle 2. While that infection remains untreated, there is an effect on getting another infection/OD. Once that infection is treated, then there is a separate effect of this infection on future infections. Therefore, this module has two multipliers: 1) one that can change the probability of an additional SDU if current SDU is untreated, and 2) one that can change the probability of a recurrent SDU (in the future) if the current SDU is fully treated and they survive it.

If an individual does not acquire an SDU in the current cycle and does not have an untreated SDU from a past cycle, they return to the simulation. If they acquire one or more SDUs, or have an untreated SDU from a past cycle, then individuals draw linkage probability to inpatient from the SDU. Linkage to inpatient depends on the linkage probability of their SDU; if an individual has more than one SDU, their linkage probability is the highest of the linkage probabilities for the SDUs they have. There remains the possibility that an individual does not link to inpatient. In the case of nonfatal OD, it implies that the OD was not severe enough to require hospitalization (or was treated in the field). In the subsequent cycle, there should not be a

© 2025 Padmanabhan P et al. *JAMA Network Open*.

flag for untreated overdose. All nonfatal overdoses are, by definition, treated so the "existing" state can only last for the cycle in which the non-fatal overdose occurs. In the case of endocarditis, the untreated flag should remain on until the person either dies or links to inpatient care and gets cured. This is because endocarditis is generally uniformly fatal if untreated. In the case of SSTI, some SSTIs can spontaneously clear (e.g., consider a pimple or slight redness around a cut). In the model, we are assuming that SSTIs being modeled are serious infections that would require hospitalization or, otherwise, ultimately lead to death. Therefore, similar to IE, the untreated SSTI flag remains on until either 1) the person dies, or 2) the person is linked to inpatient and cured, whichever occurs first. Individuals who go to the hospital will be classified as "inpatient" starting in the same cycle and will have an "in-hospital mortality." Once they leave the hospital, they are considered as having a history of infection. If an individual does not link to inpatient, they are classified as having "existing" SDU and have different risks of death (untreated mortality probabilities for each SDU). Individuals who come to the SDU module on subsequent cycles with an additional SDU (>1 SDU at a time) will have the probability of hospitalization that is equal to the highest probability of the SDUs.

Attributes that an individual can acquire in this module and should be tracked:

- Current IE
- Current SSTI
- Current OD, non-fatal
- Current OD, fatal
- History of treated IE
- History of treated SSTI
- History of treated OD

Module 3. Inpatient Hospitalization Module. One assumption of the model is that any individual that is either a) current injection drug use or b) has a current, untreated SDU is presumed to have opioid use disorder (OUD). Some sequelae of OUD are infectious and some are non-infectious (e.g., overdose).

Each individual with 1+ SDU (SDU) has a probability per cycle (7-day cycle) of presenting to an inpatient setting for their care. When individuals enter the inpatient module, the model checks their current SDU status. If they do not have a current untreated SDU or died of fatal overdose in the previous module, or they are on outpatient antibiotics, then they return to the simulation. Therefore, only those individuals with active SDU can progress through this module.

The path through the inpatient module is conditional on the SDU(s) that an individual has: nonfatal OD, IE, SSTI, or combination. The hospitalization duration for overdose is 1 cycle; the hospitalization duration for SSTI and IE are drawn stochastically from a normal distribution

© 2025 Padmanabhan P et al. *JAMA Network Open*.

with a user defined mean and standard deviation; the model allows for a maximum hospitalization to be set so that at the end of the max amount of time a person will leave the hospital. Each hospitalization is associated with a cost that is accrued in later module. The key feature of this module is that individuals may encounter a variety of in hospital services. These services are either turned on or off by the user depending on the analysis. If they are on, then individuals will have a probability of being offered and of accepting those services during their hospitalization. Some services only apply to individuals with infections (e.g., ID consultation, outpatient parenteral antibiotic treatment [OPAT]). Other services apply to people with any of the SDUs—infections or overdose—such as MOUDs, skin cleaning, etc. This is because the infection or the overdose is a sequelae of drug use (and by virtue of their OUD). Each service has an effect either within this module or elsewhere in the simulation. Each service is associated with a cost that is applied in a separate module at the end of the simulation. Individuals should be "marked" as using/receiving a service such that the cost can be tabulated in the separate module. Additionally, some of the services have an independent effect on quality of life. Similar to cost, this is applied in a separate module at the end of the simulation. Hospitalization is associated with a decreased QoL so there is a hospitalization QoL weight that can be applied in a separate module at the end of the simulation.

**Hospital-based service**	**SDU for which service applies (eligibility)**	**Effect in the model**	**Independent cost**	**Independent quality of life**
Addiction consult service	OD, SSTI, IE, combination	Change (increase) probability of linkage to outpatient addiction care*	Yes, this recurs on a weekly basis while inpatient	No
Initiation/continuation of MOUD (e.g., buprenorphine)	OD, SSTI, IE, combination	change probability of linkage to outpatient MOUD. Change probability of transitioning between injection frequency states. See treatment effect description below.	See treatment effect. Yes, recurs on a weekly basis while inpatient	Yes

© 2025 Padmanabhan P et al. *JAMA Network Open*.

Overdose education, naloxone distribution (OEND)*	OD, SSTI, IE, combination	Decrease proportion of fatal overdose for X subsequent cycles	Yes, one time per hospitalization episode	No
Skin cleaning education*	OD, SSTI, IE, combination	Decrease probability of unclean injection for X subsequent cycles after end of hospitalization	Yes, one time per hospitalization episode	No
Clean needle distribution*	OD, SSTI, IE, combination	Decrease probability of reuse needles for X subsequent cycles after end of hospitalization	Yes, one time per hospitalization episode	No
ID consult	SSTI and/or IE	Decrease in-hospital mortality from IE or SSTI during active infection;<br>Allows to access (if available, with offer/accept) OPAT	Yes, this recurs on a weekly basis while inpatient	No
OPAT**	SSTI and/or IE	Decrease hospital length of stay	Yes, one time per hospitalization episode	No

*These interventions are applied only in the last cycle of hospitalization and they will have post-treatment effective cycles drawn from a normal distribution.

**OPAT: If person has an infection and have received ID consult service and there are more than 1 cycles

MOUD continuation: instead of clearing all outpatient flags upon entering inpatient, code will first check if a person is currently in outpatient MOUD and inpatient MOUD service is available. If so, person will get automatically linked to inpatient MOUD without further draw and keeps his/her outpatient MOUD flag ON, so upon exiting inpatient he/she will go back to MOUD outpatient (without draw). However, since person is considered as inpatient, cost and mortality of

© 2025 Padmanabhan P et al. *JAMA Network Open*.

inpatient are applied (no outpatient MOUD cost). The inpatient cycles are not counted toward accumulated outpatient MOUD cycles in output but they are considered by behavior transition module when deciding MOUD effect on injection frequency transition.

In order to "leave against medical advice", ID consult and OPAT have to be on, and OPAT linkage probability has to be less than 1. In order to allow people to "leave against medical advice" with OPAT turned off, the above needs to be true, ID consult should be set to 1 (mortality benefit set to 1), OPAT linkage should be set to 0, and OPAT and ID consult costs should be set to 0.

Each individual has a probability of in-hospital mortality that is discussed in detail in the mortality module section. It is mentioned here to note that it is an attribute that an individual can acquire.

During hospitalization, individuals "carry" a flag/marker that designates them as hospitalized. While hospitalized, individuals cannot get a new SDU so they will not enter SDU module. They have an "in hospital" mortality that is conditional on the SDU for which they are hospitalized. For the duration of their hospitalization, their injection frequency is considered to be "no current" regardless of their actual status and they are not exposed to behavior transitions. The exception to this rule is as follows: In the last hospitalization cycle, individuals are exposed to behavior transitions based on their pre-hospitalization status. If they have received any intervention that would affect their behaviors (MOUD, skin cleaning education or clean needle distribution), the intervention effect will be applied to their actual or pre-hospitalization behaviors and post-treatment effective cycles will be drawn. These behavioral changes are assigned in the last inpatient cycle so that they take effect the first cycle out of inpatient. However, cost-life-mortality module still consider them as "no current".

When the inpatient hospitalization time has lapsed, then individuals move to the outpatient module. In the outpatient module, they have a probability of then linking to different types of care.

Module 4. Outpatient Care Module. There are two different ways in which an individual can enter the outpatient module. First, an individual can enter via background linkage. This means that those who are not hospitalized but "decide" to seek care can do so by entering this module. Second, an individual can enter via the inpatient module.

For individuals entering from the simulation (background). Each individual encounters the outpatient module. Individuals with a "death" flag from a previous module (fatal overdose) enter the outpatient module and immediately return to the simulation. Individuals who are currently hospitalized immediately return to the simulation. All other "ever" drug user

© 2025 Padmanabhan P et al. *JAMA Network Open*.

individuals have a probability of linking to outpatient care and progress through the outpatient module, regardless of history of SDU or drug use status. If individuals do not draw "linkage" then they return to the simulation. *For individuals entering from the inpatient module (inpatient linkage).* When the inpatient hospitalization time has lapsed, then individuals encounter a linkage probability to the outpatient module depending on inpatient services they have received.

Outpatient antibiotics. Outpatient antibiotics should be the difference between the pre-specified total duration and the duration they received in the hospital. Once the duration of antibiotics is completed, individuals with infections will be considered "cured/treated," they should unlinked from outpatient antibiotics and this service will not be available to them on subsequent cycles unless they get a new infection, enter hospital and get another OPAT. This service should have a duration associated with it. There should be a probability that individuals unlink spontaneously from outpatient antibiotics (i.e., do not finish their full duration of antibiotics). For any cycle in which an individual is maintained on antibiotics, they cannot acquire a new infection. They can, however, acquire a new infection starting in the cycle following the completion of the antibiotics.

Outpatient addiction care. Individuals will have a probability of linking to outpatient addiction care (either with or without MOUDs). One cannot be simultaneously in outpatient addiction care with MOUDs and without MOUDs (these are separate states). But Individuals can be simultaneously in outpatient addiction care (with or without MOUDs) and outpatient antibiotics.

Individuals will have a probability of unlinking from outpatient addiction care either with or without MOUDs or transitioning between MOUD states. There will be a separate probability of linking to outpatient addiction care (with or without MOUDs) for those coming from the inpatient module and those coming from the simulation (spontaneous linkage/background linkage). There are different linkage probabilities for the following groups:

1. Individuals who have received inpatient addiction care but did not get MOUD
    - They will have a probability of linking to addiction care (w/wo MOUD) and then conditional on linking, they have another probability to determine the chance of getting MOUD.
2. Individuals who have received inpatient addiction care and got MOUD
    - Model assumes if someone is already on MOUD and if they link to outpatient addiction care, they will get MOUD. So they only have 1 probability to determine whether they will link to outpatient addiction care with MOUD or not.
3. Individuals who did not receive inpatient addiction care but got inpatient MOUD
    - Same assumption as (2) but the value of the probability may differ.
4. Individuals who did not receive any relevant inpatient services or individuals coming from the background (no hospitalization)
    - same as (1) but the value of the probability may differ.

© 2025 Padmanabhan P et al. *JAMA Network Open*.

If an individual is in outpatient addiction care and acquires an infection (SSTI or IE) they will automatically be linked to inpatient care in the next cycle. In this case, they will unlink from outpatient care and all outpatient related flags/cycles will be cleared.

If someone is linked to both outpatient antibiotics and outpatient addiction care (w/wo MOUD), if they unlink from antibiotics, they will also automatically unlink from addiction care. Unlinking will take effect in the next cycle.

**Outpatient service**	**Eligibility**	**Effect in the model, while linked**	**Independent cost**	**Independent quality of life**
Outpatient antibiotics (IV or oral)	Active SSTI, active IE, OPAT available inpatient, OPAT accepted and linked	Prevent new infection while on antibiotics. No infection related cost, utility and mortality	Yes	No
Outpatient addiction with MOUD	Any ever IDU in the simulation not actively hospitalized	Change (decrease) probability of unclean injection; change (decrease) needle sharing, change (increase) probability of moving to lower frequency state and change (decrease) probability of moving out of no/low frequency state. See treatment effect document.	Yes	Yes

© 2025 Padmanabhan P et al. *JAMA Network Open*.

Outpatient addiction without MOUD	Any ever IDU individual in the simulation not actively hospitalized	Change (decrease) probability of unclean injection; change (decrease) needle sharing	Yes	No

Module 5: Behavioral Transitions Module. Following the inpatient and outpatient modules, individuals will move to the behavioral transitions module. Individuals may also enter this module "from the simulation." This latter represents the ability of someone to change their behaviors organically (without interventions). This is the module in which they can move between high frequency, low frequency, and no current use states, move from never and ever idu, move between skin cleaning and not skin cleaning states, and move between sharing needles and not sharing needles states. There will be a prior probability of movement between states (status quo) and various "flags" acquired throughout the model progression that will impact certain probabilities. These have been outlined in various other module descriptions but will also be outlined below.

Treatment Effects: The primary driver of morbidity and mortality in the module is the injection frequency. High frequency individuals are at higher risk than low frequency injectors of sequelae of drug use (SDUs), which include overdose, skin/soft tissue infections, and endocarditis in this model. All persons who are "ever" injectors have the possibility of moving to a higher or lower injection frequency state (depending on their current state) or staying in their current state per cycle. For instance, a high frequency injector may remain as a high frequency injector or may move to low frequency or no current use states. There are a few ways that the injection frequency can be modified in the model. <u>In brief, however, only hospitalization and MOUDs can change injection frequency in the model.</u>

Mechanisms by which transitions between injection frequency states are changed:

1) **Hospitalization.** If an individual becomes hospitalized for an SDU, then cost, life, and mortality modules will consider their injection state as "no current use" and behavior module returns them to simulation (without any changes) during their hospitalization cycles. The only exception is the last hospitalization cycles in which behavior module considers them as their pre-hospitalization behavior states and updates their behaviors based on inpatient services received (if none, then no intervention values are used).

© 2025 Padmanabhan P et al. *JAMA Network Open*.

2) **Outpatient MOUD initiation.** Individuals have the possibility of initiating MOUDs in the model by linking to outpatient care (linkage to outpatient care comes either via "background linkage" or linkage following hospitalization). When an individual initiates MOUDs in the outpatient setting, there is an immediate treatment effect which lasts for 4 cycles (4 weeks). During those 4 cycles, there is a high probability of moving to no active use state and a low probability of moving to or back to a higher drug use state (relapsing). After the treatment initiation effect but while still on MOUDs, they encounter a probability of remaining in the no current drug use state or moving to a lower drug use state that is different than the "no MOUD" status (since the longer a person remains on treatment, the more likely they are not to relapse) and "immediate treatment effect".
3) **Inpatient MOUD initiation.** In some scenarios, individuals have the opportunity to initiate MOUDs during their hospitalization for SDU (i.e., when certain inpatient services are available). When an individual initiates MOUDs in the inpatient module and then links to outpatient care, the individual automatically links to MOUDs in outpatient (ie if a person links from inpatient with MOUD to outpatient, they cannot link to outpatient without MOUD as this doesn't make sense).If an individual unlinks from inpatient care and does not link to outpatient care, they discontinue MOUD.
4) **Behavioral transitions with MOUD**. When an individual links to MOUD, whether inpatient or outpatient, there is a treatment effect which changes the probability of moving to different drug use states (higher probability of staying off drugs or moving to a lower drug use state, lower probability of moving to a higher drug use state). Eventually, the model should be able to differentiate between the 4 week "initiation effect", and the post-4 week "maintenance effect", which have different relapse rates.

Mechanisms by which skin cleaning is modified:

Proper skin cleaning can be modified in the model via two mechanisms. However, both of these influence the transition probabilities.

1. **Outpatient addiction care.** Risk reduction is a component of addiction care, this includes skin cleaning education. This increases the probability of moving to skin cleaning status. The effect is applied as long as a person is engaged in outpatient addiction care and for a set duration of time afterwards. Durations are drawn from a normal distribution and have a maximum value.
2. **Inpatient skin cleaning intervention.** Simply put, if a skin cleaning intervention is available and an individual receives the intervention, then this increases their probability of being a "skin cleaner". This probability is applied in the last hospitalization cycle and until post-treatment effective cycles, which are drawn from a normal distribution are in effect.

Mechanisms by which cleaning needles/not reusing is modified:

Proper needle sharing can be modified in the model via two mechanisms. However, both of these influence the transition probabilities.

1. **Outpatient addiction care.** Risk reduction is a component of addiction care, this can include needle distribution. This increases the probability of moving to non needle sharing status. The effect is applied as long as a person is engaged in outpatient addiction care and for a set duration of time afterwards. Durations are drawn from a normal distribution and have a maximum value.
2. **Inpatient clean needle distribution.** Simply put, if a needle distribution is available and an individual receives the intervention, then this increases their probability of being a "non-sharer". This probability is applied in the last hospitalization cycle and until post-treatment effective cycles(which are drawn from a normal distribution) are in effect.

Module 6: Mortality Module. The mortality module is more than mortality. It includes costs and quality of life adjustments as well.

Mortality. There are two places in the model that an individual can die: fatal overdoses in the SDU module and in the mortality module. To review, in the SDU module, an individual draws a combined probability of all types overdose which is stratified by injection frequency (high and low frequency). From that combined probability, an individual can draw either a fatal or non-fatal overdose. If an individual draws a fatal overdose, then go through the remainder of the cycle with a "fatal OD" flag up which does not allow them to get any further interventions, collect additional costs, change their behavior status, etc., however, they will accumulate the background cost and utility of that cycle. As such, the background mortality in the mortality module should exclude overdose mortality.

The background mortality risk will be an age and sex adjusted mortality probability (excluding fatal overdose). This will be derived from the NVSS database, with overdose removed from this data. Any person aged 100 years will automatically be set to "dead". There are a number of occurrences in the model that can impact the weekly risk of mortality. First, individuals who are hospitalized for an SDU (non-fatal overdose, SSTI, or endocarditis) have an increased risk of death. If the inpatient individual further gets an ID consult, their infection inpatient mortality rate is augmented by an ID consult mortality multiplier (ID consult will not affect overdose mortality). Second, individuals who have an untreated skin and soft tissue infection or untreated infective endocarditis will have an increased risk of death. These risks are input as probabilities (and converted to rates by the model) which are then ***added*** to the background mortality at the end of each cycle. Once a patient is cured of their infection their SDU flags are removed and their mortality goes back to background mortality. The mortality risk only applies for each cycle that they have that risk. For example, a person gets endocarditis and does not present to inpatient care during a cycle. Then they have an "existing endocarditis" flag that the end of the cycle should prompt the rate of death for untreated endocarditis to be added to the background mortality. On cycles 2-5 that same individual, however, is hospitalized and being treated for their endocarditis. For those cycles, the "untreated" SDU flag drops off but they get

an “in-hospital for endocarditis” flag such that the in-hospital endocarditis mortality rate is added to their background mortality each cycle. On cycle 6, this person leaves the inpatient setting (completes treatment) so all flags are, therefore, off and at the end of that cycle they get only background mortality.

Individuals on outpatient antibiotics are not considered as being in inpatient or having an untreated infection, therefore, they do not have additional mortality due to infection.

For this version of the model, we do not include an additional mortality risk for being an active drug user since most of that risk will be folded into overdose and other SDUs.

Cause of death as an output: In the model, individuals can die of background causes or as a direct result of their injection drug use. Direct causes of injection drug use include:

- Overdose (combination of fatal overdose/ hospitalized and nonfatal OD that dies in the hospital)
- endocarditis (combination of hospitalized and non-hospitalized)
- SSTI (combination of hospitalized and non-hospitalized)

Aside from fatal overdose, all of the other causes of death get added to the background mortality as outlined above. For instance, an individual’s weekly probability of death (conditional on not dying of a fatal overdose) may be p_d and they may have endocarditis which increases their risk of death by x. The individual’s weekly risk of death is, therefore, *the sum of the rates converted to a probability.* However, as an output, we need to be able to determine the attributable cause of death (this person may have died of endocarditis OR background causes). To do this, we use the sum of the rates as the denominator and the individual mortality risk (rates) as the numerator in drawing the cause of death. Important for consistency, the input parameters are probabilities and therefore all rates are calculated in the model.

© 2025 Padmanabhan P et al. *JAMA Network Open*.

Parameterization

We used two independent approaches to arrive at an estimate of 9,697 PWID in Denver. First, we applied nationwide estimates of the prevalence of current injection drug use to Denver's Census-derived population.[3,4] Second, we assumed that the national incidence of fatal opioid overdose in 2022 was equivalent to that of Denver, and divided the number of recorded fatal overdoses in Denver by this incidence rate to estimate a denominator of people at risk of opioid overdose.[5,6] These methods resulted in estimates of 9,754 and 9,640, respectively, and were averaged to 9,697.

We derived cohort-specific input parameters for key behavioral variables representing the effects of SSPs and OPCs. 2022 NHBS data sampling a population of PWID in Denver was stratified by past-year SSP usage to derive demographics, injection behaviors, and MOUD initiation parameters for cohorts 1 and 2. For the SSP-using cohort (cohort 2), we also applied Nassau et. al.'s estimate of the odds of syringe sharing among PWID in Denver accessing an SSP compared to those who did not (OR=0.23) to parameterize behavior transition probabilities.[7] This is representative of simulated individuals being able to receive the protective benefits of an SSP throughout the simulated period.

To parameterize the cohort of PWID using an OPC (cohort 3), we first replicated the parameters of the SSP-using cohort (cohort 2), and applied changes to five parameters: (1) initial and (2) transition probabilities of syringe sharing, (3) initial and (4) transition probabilities of skin cleaning, and (5) weekly probability of overdose. Suen et. al. found that the odds of syringe sharing among OPC users was 0.17 (95% CI: 0.03, 1.02) times that of non-OPC users[8], and Stoltz et. al. found that the odds of cleaning the injection site prior to injection among OPC users was 2.85 times (95% CI: 2.09, 3.87) that of non-OPC users.[9] Integrating public data from OnPoint, an OPC in New York City, and overdose data from CDC, we found that overdose reversals at OnPoint represented approximately 1.92% of fatal ODs in the city of New York between December 2021 and July 2023.[10,11] Therefore, we reduced the probability of overdose by this amount. The probability of initiating MOUD was the same between cohort 2 and cohort 3.[12] Input parameter values are provided in Table S1.

Calibration

Calibration targets for select outcomes were collected from literature based on cohort studies and surveillance of PWID in the United States, Canada, and Australia. In case of multiple sources of data reporting differing estimates, more recent data and those evaluating populations in the United States were preferred. Calibration processes involved iteratively adjusting input parameters within reported ranges until model outputs were simultaneously either within calibration target ranges or within 15% of point estimates. Input parameters represented national (US) estimates of PWID. Calibration output is reported in Table S2.

© 2025 Padmanabhan P et al. *JAMA Network Open*.

Validation

In addition to calibration of our model to national data, we assessed external validity by comparing modeled fatal overdose outcomes to data on fatal overdoses in Denver in 2022 collected by the Colorado Department of Public Health and Environment.[6] With 241 fatal overdoses and our estimated population of 9,697 PWID (see manuscript for calculations), the fatal overdose rate in Denver in 2022 was approximately 24.85 per 1,000 PWID. Our modeled outcome, after weighting non-SSP and SSP users, was 24.68 per 1,000 PWID, an approximate 0.7% difference.

Sensitivity analyses

We conducted two deterministic sensitivity analyses (DSAs) reflecting a "best case" and "worst case" scenario. In the "best case" scenario, the initial probability and transition probabilities of skin cleaning associated with OPC use was calculated based on the upper end of the odds ratio identified by Stoltz et. al., while the odds of syringe sharing was taken from the lower end of the odds ratio identified by Suen et. al. The opposite ends of each range were used to generate "worst case" input parameters. We also conducted two DSAs utilizing the "best case" odds for skin cleaning and "worst case" odds for needle sharing, and vice versa.

Finally, we conducted a DSA in which we assumed a greater reduction in fatal overdoses associated with OPCs (a 5.91% reduction in the probability of overdose) than in the primary analysis (a 1.92% reduction). To derive this parameter, we used estimates from Rammohan et. al., who found that OPCs were associated with a reduction in approximately 2 fatal overdoses within a square mile of OPCs per 100,000 residents of Toronto.[41] This estimate is more liberal than the primary analysis, though likely conservative in that it assumes no reduction in fatal overdoses associated with OPCs beyond a mile radius. Extrapolating to Denver's population in 2022 (approximately 713,000) and dividing by the number of fatal overdoses in that year (241) gives a 5.91% reduction.

Changes in all outcomes compared to the Status Quo for all DSAs are provided in Tables S3-S4.

Alternative scenarios

OPC-Community Health Center model

We considered two scenarios involving the implementation of an OPC using a "Community Health Center" (OPC-CHC) model. In the first scenario ("OPC-CHC with Current Reach"), the OPC did not increase overall utilization of harm reduction services (i.e. the population of PWID using the OPC was drawn from the existing pool of PWID using an SSP). In the second scenario ("OPC-CHC with Expanded Reach"), the OPC was able to attract PWID

© 2025 Padmanabhan P et al. *JAMA Network Open*.

who do not currently use an SSP. These mirror scenarios 2 and 3 (*OPC with Current Reach* and *OPC with Expanded Reach*, respectively) from the primary analysis.

The following model input parameters were influenced by the OPC-CHC, and are compared to the cohort who did not utilize any harm reduction and the corresponding cohort of PWID using an OPC under a harm reduction model ("OPC-HR") in the primary analysis:

Compared to the *Status Quo*, the *OPC-CHC with Current Reach* scenario resulted in a decrease in IE incidence of -3.3% (95% CrI: -3.8, 0.7), an increase in SSTI incidence of 0.8% (95% CrI: -0.4, 2.7), a decrease in fatal overdoses of -0.8% (95% CrI: -2.2, 2.0) and a decrease in nonfatal overdoses of -0.8% (95% CrI: -1.6, 0.9) while the *OPC-CHC with Expanded Reach* scenario resulted in a decrease in IE incidence of -27.5% (95% CrI: -29.6, 2.1), an increase in SSTI incidence of 5.3% (95% CrI: -3.3, 17.9), a decrease in fatal overdoses of -6.0% (95% CrI: -14.8, 13.1), and a decrease in nonfatal overdoses of -2.8% (95% CrI: -4.6, 14.0).

OPC-CHC with Current Reach was associated with an increase in hospitalizations of 1.5% (95% CrI: 0.2, 3.5) and *OPC-CHC with Expanded Reach* was associated with an increase in hospitalizations of 10.6% (95% CrI: 1.8, 24.2) over 10 years. The mortality rate decreased by -2.5% (95% CrI: -2.9, -0.6) and -19.4% (95% CrI: -21.2, -5.6) in *OPC-CHC with Current Reach* and *OPC-CHC with Expanded Reach* scenarios respectively.

Compared to an average cost per person of $387,700 under the *Status Quo*, the *OPC-CHC with Current Reach* scenario had a slightly higher cost of $390,400 per person, while the *OPC-CHC with Expanded Reach* scenario was more expensive at $408,300 per person. The *OPC-CHC with Current Reach* scenario was associated with 0.028 increased life years, while the *OPC-CHC with Expanded Reach* scenario was associated with 0.21 increased life years.

In scenarios in which an OPC was operating under a community health center model, we found an increase in infections and hospitalizations compared to the status quo, but a greater decrease in mortality compared to the *Status Quo* and the OPC-HR scenarios. The increase in infections and hospitalizations could be attributable to overall longer life spans and greater life-years during which PWUD could accumulate infections, while decreased mortality could be attributable to an increase in initiations of MOUD.

Harm Reduction abolished

We considered a scenario in which all existing formal harm reduction services in Denver (i.e. Syringe service programs) were shut down ("No Harm Reduction"). In this scenario, 100% of PWID in Denver were assigned the characteristics of cohort 1 in the primary analysis (those who did not utilize any harm reduction services).

© 2025 Padmanabhan P et al. *JAMA Network Open*.

Under the *No Harm Reduction* scenario, the model predicted an increase in SSTI incidence of 4.6% (95% CrI: -2.4, 15.0), an increase in IE incidence of 41.7% (95% CrI: 7.4, 52.2), an increase in fatal overdoses of 1.3% (95% CrI: -20.6, 17.7), and a decrease in nonfatal overdoses of -26.1% (95% CrI: -36.4, -18.1). *No Harm Reduction* was associated with a decrease in hospitalizations of -3.7% (95% CrI: -12.1, 5.8), while mortality increased by 15.9% (95% CrI: -0.2, 25.3). The decrease in projected hospitalizations in the *No Harm Reduction* scenario is primarily due to an increase in mortality. In this scenario, PWID often died of fatal overdose or severe infection before accessing care. Average discounted costs per person in this scenario were $369,000 per person (95% CrI: $342,100, $385,900), compared to $387,700 (95% CrI: $358,800, $401,200) per person under the *Status Quo*. A higher proportion of costs were attributable to hospitalization, with 86.7% compared to 83.1% under the *Status Quo* scenario.

Among PWID, allowing bacterial SSTIs like cellulitis to advance untreated increases the risk of developing more serious infections including endocarditis.[43] This is borne out in modeled outcomes of dramatic increases in IE incidence and hospitalization costs in scenarios in which harm reduction services are abolished.

© 2025 Padmanabhan P et al. *JAMA Network Open*.

eTable 1. Input Parameter Values Across Simulated Cohorts

Parameter	Estimate, No harm reduction utilization (cohort 1)	Estimate, SSP utilization only (cohort 2)	Estimate, OPC utilization (cohort 3)	Reference
**Population**				
Mean age (years)	42.3	41.1	41.1	[13]
% male	68.2	75.1	75.1	[13]
% high-frequency injection	47.9	76.3	76.3	[13]
% low-frequency injection	16.8	13.1	13.1	[13]
% no current injection	35.3	10.6	10.6	[13]
% unsterile injection	29.0	12.4	5.7	[9,13]
% sharing needles	15.4	13.9	2.7	[8,12,13]
**Sequelae of drug use**				
Probability of overdose				
High-frequency injection	0.0130	0.0130	0.01275	[13,14]
Low-frequency injection	0.005	0.005	0.0049	[13,14]
Probability of fatal OD, conditional on OD	0.062	0.048	0.048	[5]
Probability of SIRI (composite; probability is stratified by injection behaviors)	0.008	0.008	0.008	[4,15]
Probability of IE, conditional on infection	0.0300	0.0300	0.0300	[15,16]
**Inpatient and outpatient services**				
Probability of MOUD initiation, background	0.0121	0.0171	0.0171	[12,13]
Probability of inpatient linkage for SSTI	0.0030	0.0030	0.0030	[17,18]

© 2025 Padmanabhan P et al. *JAMA Network Open*.

Probability of inpatient linkage for IE	0.2	0.2	0.2	[19]
Probability of inpatient linkage for OD	0.135	0.135	0.135	[20]
**Behavioral transitions**				
Probability of transition, not skin cleaning to skin cleaning (not on MOUD)	0.0145	0.0145	0.0403	[26]
Probability of transition, not skin cleaning to skin cleaning (on MOUD)	0.0152	0.0152	0.0421	[26]
Probability of transition, skin cleaning to not skin cleaning (not on MOUD)	0.0361	0.0361	0.0361	[26]
Probability of transition, skin cleaning to not skin cleaning (on MOUD)	0.0258	0.0258	0.0258	[26]
Probability of transition, not sharing needles to sharing needles (not on MOUD)	0.0235	0.0235	0.0235	[16]
Probability of transition, not sharing needles to sharing needles (on MOUD)	0.0194	0.0194	0.0194	[16]
Probability of transition, sharing needles to not sharing needles (not on MOUD)	0.0156	0.0646	0.0855	[16]
Probability of transition, sharing needles to not	0.0200	0.0815	0.1072	[16]

sharing needles (on MOUD)				
**Healthcare costs (2022 US$)**				
SSTI hospitalization	20227.13	20227.13	20227.13	[21]
IE hospitalization	24581.57	24581.57	24581.57	[21,22]
OD hospitalization	16175.04	16175.04	16175.04	[23]
Addiction consult	256.38	256.38	256.38	[24,25]
ID consult	922.24	922.24	922.24	[24]
OPAT	178.89	178.89	178.89	[26]
MOUD	49.71	49.71	49.71	[27]
Antibiotics	278.58	278.58	278.58	[26]
Addiction care with MOUD	135.08	135.08	135.08	[24,28]
Addiction care without MOUD	92.30	92.30	92.30	[24,28]

© 2025 Padmanabhan P et al. *JAMA Network Open*.

eTable 2. Calibration Targets and Modeled Output

Outcome	Target value/range*	Model output	Difference	References
Nonfatal OD incidence (per 100 person-years)	33.2 – 41.0	38.2	-	[5]
Nonfatal OD one-year prevalence	18.7% - 27.3%	26.8%	-	[29]
Fatal OD incidence (per 100 person-years)	2.0 – 2.5	2.14	-	[5]
SSTI one-year prevalence	23.7% - 47.1%	25.2%	-	[4,16,17]
SSTI hospitalization one-year recurrence	16%	14.56%	-9.0%	[30]
IE one-year prevalence	1.3% - 2.6%	1.56%	-	[16,17]
IE hospitalization incidence (per 100 person-years)	0.65 – 0.8	1.07	33.8%**	[15,31,32]
IE hospitalization one-year recurrence	12% - 22.3%	13.1%	-	[33]
IE case fatality rate	32.7%	34.3%	4.9%	[34]
Average MOUD duration (weeks)	13.5 - 21.4	15.4	-	[35,36]
IE in-hospital mortality	11.5%	10.7%	-7.0%	[37]
SSTI in-hospital mortality	0.8%	0.79%	-1.3%	[37]
Proportion of hospitalizations for both SSTI and IE simultaneously	7.4%	6.4%	-13.5%	[37]
All-cause mortality (per 100 person-years)	3.8	3.48	-8.4%	[38-40]

*For incidence rates, target values from literature were multiplied by 0.81, as 19% of our model cohort consisted of PWID who had a history of injecting but were not actively injecting at the beginning of the simulation, and were therefore much less likely to experience sequelae of drug use. Prevalence and mortality targets were taken directly from literature, as the aforementioned simulated individuals had the ability to become active injectors during the simulation.

**While model output for IE hospitalization incidence was higher than the target range, IE prevalence and mortality were both within accepted ranges, with IE prevalence towards the lower bound of the target range. The best available calibration target data for IE hospitalization was collected prior to 2018, while injection-related IE hospitalization incidence has been trending upward due to increased prevalence and frequency of injection drug use and has thus likely increased since that time.

© 2025 Padmanabhan P et al. *JAMA Network Open*.

eTable 3. Changes in Outcomes in DSAs under OPC with Current Reach Scenario

	IE incidence	SSTI incidence	Fatal overdoses	Nonfatal overdoses	Hospitalizations	Mortality
Best case	-5.0%	-3.4%	1.3%	1.4%	-2.7%	-1.1%
Worst case	4.3%	2.1%	-1.1%	-1.1%	1.5%	0.7%
High needle sharing, high skin cleaning	2.6%	1.3%	-0.8%	-0.8%	1.0%	0.4%
Low needle sharing, low skin cleaning	-4.3%	-2.9%	1.0%	1.1%	-2.3%	-1.0%
Greater reduction in overdoses	-2.5%	-1.5%	-0.03%	0.01%	-1.3%	-0.8%

© 2025 Padmanabhan P et al. *JAMA Network Open*.

eTable 4. Changes in Outcomes in DSAs under OPC with Expanded Reach Scenario

	IE incidence	SSTI incidence	Fatal overdoses	Nonfatal overdoses	Hospitalizations	Mortality
Best case	-39.1%	-24.4%	9.1%	12.3%	-18.5%	-9.2%
Worst case	25.7%	13.7%	-8.0%	-5.2%	11.2%	3.5%
High needle sharing, high skin cleaning	14.0%	8.7%	-6.0%	-2.8%	7.3%	1.1%
Low needle sharing, low skin cleaning	-34.7%	-20.4%	7.2%	10.5%	-15.4%	-8.4%
Greater reduction in overdoses	-21.9%	-11.2%	-0.3%	2.7%	-8.7%	-7.3%

Scenarios in which OPCs were associated with decreases in needle sharing resulted in decreases in infections, hospitalizations, and mortality and increases in overdoses, while scenarios in which skin cleaning increased but the odds of needle sharing among OPC users was 1.02 times the odds of needle sharing among non-OPC users, the “worst case” odds identified by Suen et. al., resulted in opposite trends. When OPCs have a greater impact on overdoses, infection incidence, hospitalizations, and mortality decrease. Offsetting changes in infection and overdose incidence may be indicative of competing risks. The wide variation in DSA outcomes suggests a need for greater data collection on how OPCs affect injection behavior. The “standard of care” comparison group in the status quo includes a large proportion of SSP users. Because the protective effects of SSPs are more well-specified, deterministic analyses utilizing the more uncertain ranges associated with OPCs may lead to wider estimates.

© 2025 Padmanabhan P et al. *JAMA Network Open*.

eTable 5. Selected model inputs for analysis of clinical impacts and cost-effectiveness of OPC-CHC implementation

Parameter	Estimate (Range), No harm reduction utilization	Estimate (Range), OPC-HR utilization	Estimate (Range), OPC-CHC utilization	Reference for OPC-CHC data
% unsterile injection	29.0	5.7	11.4	[42]
% sharing needles	15.4	2.7	5.6	[42]
Probability of MOUD initiation, background	0.0121	0.0171	0.0647	[42]

eTable 6. Selected Clinical Outcomes by Scenario

Strategy	Estimate (95% CrI), SSTI incidence*	Estimate (95% CrI), IE incidence*	Estimate (95% CrI), SSTI, IE, and OD hospitalization rate*	Estimate (95% CrI), All-cause mortality rate*
Status Quo (ref.)	14184 (9993, 16073)	1241 (1061, 3603)	15094 (11865, 17586)	2421 (2162, 3723)
OPC with Current Reach	13960 (9843, 15831)	1210 (1034, 3520)	14903 (11706, 17390)	2406 (2153, 3710)
OPC with Expanded Reach	12550 (8832, 14330)	968 (837, 2929)	13816 (10773, 16304)	2280 (2070, 3591)

*Rates per 9,697 PWID over a 10-year period (2022-2032)

Notes:

Abbreviations:

OPC=overdose prevention center

SSTI=skin and soft tissue infections

IE=infective endocarditis

OD=overdose

PWID=people who inject drugs

© 2025 Padmanabhan P et al. *JAMA Network Open*.

eReferences

1. Barocas JA, Nall SK, Axelrath S, et al. Population-level health effects of involuntary displacement of people experiencing unsheltered homelessness who inject drugs in US cities. *JAMA*. 2023;329(17):1478-1486.
2. Barocas JA, Savinkina A, Adams J, et al. Clinical impact, costs, and cost-effectiveness of hospital-based strategies for addressing the US opioid epidemic: a modelling study. *The Lancet Public Health*. 2022;7(1):e56-e64. doi:10.1016/s2468-2667(21)00248-6
3. Bradley H, Hall EW, Asher A, et al. Estimated number of people who inject drugs in the United States. *Clin Infect Dis*. 2023;76(1):96-102.
4. Degenhardt L, Webb P, Colledge-Frisby S, et al. Epidemiology of injecting drug use, prevalence of injecting-related harm, and exposure to behavioural and environmental risks among people who inject drugs: a systematic review. *The Lancet Global Health*. 2023;11(5):e659-e672. doi:10.1016/s2214-109x(23)00057-8
5. Shealey JY, Hall EW, Pigott TD, et al. Systematic review and meta-analysis to estimate the burden of non-fatal and fatal overdose among people who inject drugs living in the U.S. and comparator countries: 2010 – 2023. Cold Spring Harbor Laboratory; 2024.
6. Drug Overdose in Colorado Story. Colorado Department of Public Health and Environment. Accessed February 21, 2025. https://cohealthviz.dphe.state.co.us/t/PSDVIP-MHPPUBLIC/views/Historicdrugoverdosedashboard0021/DrugsinCOStory?%3Aembed=y&%3AisGuestRedirectFromVizportal=y
7. Nassau T, Al-Tayyib A, Robinson WT, Shinefeld J, Brady KA. The Impact of Syringe Services Program Policy on Risk Behaviors Among Persons Who Inject Drugs in 3 US Cities, 2005-2015. *Public Health Reports®*. 2020;135(1_suppl):138S-148S. doi:10.1177/0033354920930137
8. Suen LW, Davidson PJ, Browne EN, Lambdin BH, Wenger LD, Kral AH. Effect of an Unsanctioned Safe Consumption Site in the United States on Syringe Sharing, Rushed Injections, and Isolated Injection Drug Use: A Longitudinal Cohort Analysis. *JAIDS J Acquired Immune Defic Syndromes*. 2022;89(2):172-177. doi:10.1097/qai.0000000000002849
9. Stoltz J-A, Wood E, Small W, et al. Changes in injecting practices associated with the use of a medically supervised safer injection facility. *Journal of Public Health*. 2007;29(1):35-39. doi:10.1093/pubmed/fdl090
10. Soto E. OnPoint NYC, Operator of the Nation's First Overdose Prevention Centers, Announces That It Has Intervened In Over 1,000 Overdoses. Updated August 9, 2023. Accessed May 6, 2025. https://onpointnyc.org/intervened-in-over-1000-overdoses/
11. Statistics NCfH. Provisional Drug Overdose Death Counts. Centers for Disease Control and Prevention. https://www.cdc.gov/nchs/nvss/vsrr/drug-overdose-data.htm
12. Lalanne L, Roux P, Donadille C, et al. Drug consumption rooms are effective to reduce at-risk practices associated with HIV/HCV infections among people who inject drugs: Results from the COSINUS cohort study. *Addiction*. 2024;119(1):180-199. doi:10.1111/add.16320
13. Al-Tayyib A. Data from: Risk Education Aimed at Community Health. 2022.
14. McAteer JM, Mantha S, Gibson BE, et al. NYC's Overdose Prevention Centers: Data from the First Year of Supervised Consumption Services. *NEJM Catalyst*. 2024;5(5)doi:10.1056/cat.23.0341
15. See I, Gokhale RH, Geller A, et al. National Public Health Burden Estimates of Endocarditis and Skin and Soft-Tissue Infections Related to Injection Drug Use: A Review. *The Journal of Infectious Diseases*. 2020;222(Supplement_5):S429-S436. doi:10.1093/infdis/jiaa149

© 2025 Padmanabhan P et al. *JAMA Network Open*.

16. Larney S, Peacock A, Mathers BM, Hickman M, Degenhardt L. A systematic review of injecting-related injury and disease among people who inject drugs. *Drug and alcohol dependence*. 2017;171:39-49.
17. Figgatt MC, Salazar ZR, Vincent L, et al. Treatment experiences for skin and soft tissue infections among participants of syringe service programs in North Carolina. *Harm Reduction Journal*. 2021;18(1)doi:10.1186/s12954-021-00528-x
18. Curtis SJ, Colledge-Frisby S, Stewardson AJ, et al. Prevalence and incidence of emergency department presentations and hospital separations with injecting-related infections in a longitudinal cohort of people who inject drugs. *Epidemiology and Infection*. 2023;151:1-27. doi:10.1017/s0950268823001784
19. N'Guyen Y, Duval X, Revest M, et al. Time interval between infective endocarditis first symptoms and diagnosis: relationship to infective endocarditis characteristics, microorganisms and prognosis. *Ann Med*. 2017;49(2):117-125. doi:10.1080/07853890.2016.1235282
20. Data from: Drug Overdose Surveillance and Epidemiology (DOSE) System: Nonfatal Overdose Emergency Department and Inpatient Hospitalization Discharge Data. 2023.
21. Coye AE, Bornstein KJ, Bartholomew TS, et al. Hospital Costs of Injection Drug Use in Florida. *Clin Infect Dis*. 2021;72(3):499-502. doi:10.1093/cid/ciaa823
22. Fleischauer AR, Laura; Rhea, Sarah; Barnes, Erin. *Hospitalizations for Endocarditis and Associated Health Care Costs*

Among Persons with Diagnosed Drug Dependence —

North Carolina, 2010–2015. Vol. 66. 2017. *Morb Mortal Weekly Rep*. June 9, 2017. https://www.cdc.gov/mmwr/volumes/66/wr/pdfs/mm6622a1.pdf
23. Association HFM. Annual Hospital Costs for Opioid Overdose Patients Approaches $2 Billion. Accessed March 6, 2025. https://www.hfma.org/accounting-and-financial-reporting/cost-of-care/63299/
24. Services CfMM. Physician Fee Schedule. Centers for Medicare & Medicaid Services. 2025. https://www.cms.gov/medicare/payment/fee-schedules/physician
25. Affairs USDoV. Pharmaceutical pricing- VA National Acquisition Center programs. U.S. Department of Veterans Affairs. Accessed January 20, 2022. https://www.fss.va.gov/index.asp
26. Krah NM, Bardsley T, Nelson R, et al. Economic Burden of Home Antimicrobial Therapy: OPAT Versus Oral Therapy. *Hospital Pediatrics*. 2019;9(4):234-240. doi:10.1542/hpeds.2018-0193
27. Services CfMM. Laboratory Fee Schedule. Centers for Medicare & Medicaid Services. 2022. https://www.cms.gov/medicare/payment/fee-schedules/clinical-laboratory-fee-schedule-clfs
28. Toseef MU, Durfee J, Podewils LJ, et al. Total cost of care associated with opioid use disorder treatment. *Prev Med*. 2023;166:107345. doi:10.1016/j.ypmed.2022.107345
29. Colledge S, Peacock A, Leung J, et al. The prevalence of non-fatal overdose among people who inject drugs: A multi-stage systematic review and meta-analysis. *Int J Drug Policy*. 2019;73:172-184. doi:10.1016/j.drugpo.2019.07.030
30. Brothers TD, Bonn M, Lewer D, et al. Social and structural determinants of injection drug use-associated bacterial and fungal infections: A qualitative systematic review and thematic synthesis. *Addiction*. 2023;118(10):1853-1877. doi:10.1111/add.16257
31. Schranz A, Barocas JA. Infective endocarditis in persons who use drugs: epidemiology, current management, and emerging treatments. *Infectious Disease Clinics*. 2020;34(3):479-493.

© 2025 Padmanabhan P et al. *JAMA Network Open*.

32. Kadri AN, Wilner B, Hernandez AV, et al. Geographic Trends, Patient Characteristics, and Outcomes of Infective Endocarditis Associated With Drug Abuse in the United States From 2002 to 2016. *Journal of the American Heart Association*. 2019;8(19)doi:10.1161/jaha.119.012969
33. Rodger L, Shah M, Shojaei E, Hosseini S, Koivu S, Silverman M. Recurrent Endocarditis in Persons Who Inject Drugs. *Open Forum Infectious Diseases*. 2019;6(10)doi:10.1093/ofid/ofz396
34. Rodger L, Glockler-Lauf SD, Shojaei E, et al. Clinical Characteristics and Factors Associated With Mortality in First-Episode Infective Endocarditis Among Persons Who Inject Drugs. *JAMA Network Open*. 2018;1(7):e185220. doi:10.1001/jamanetworkopen.2018.5220
35. Wakeman SE, Larochelle MR, Ameli O, et al. Comparative Effectiveness of Different Treatment Pathways for Opioid Use Disorder. *JAMA Network Open*. 2020;3(2):e1920622. doi:10.1001/jamanetworkopen.2019.20622
36. Morgan JR, Walley AY, Murphy SM, et al. Characterizing initiation, use, and discontinuation of extended-release buprenorphine in a nationally representative United States commercially insured cohort. *Drug and Alcohol Dependence*. 2021;225:108764. doi:10.1016/j.drugalcdep.2021.108764
37. Gomes T, Kitchen SA, Tailor L, et al. Trends in Hospitalizations for Serious Infections Among People With Opioid Use Disorder in Ontario, Canada. *J Addict Med*. 2022;16(4):433-439. doi:10.1097/adm.0000000000000928
38. Sun J, Mehta SH, Astemborski J, et al. Mortality among people who inject drugs: a prospective cohort followed over three decades in Baltimore, MD, USA. *Addiction*. 2022;117(3):646-655. doi:10.1111/add.15659
39. Veldhuizen S, Callaghan RC. Cause-specific mortality among people previously hospitalized with opioid-related conditions: a retrospective cohort study. *Ann Epidemiol*. 2014;24(8):620-624. doi:10.1016/j.annepidem.2014.06.001
40. Suzuki J, Johnson JA, Montgomery MW, et al. Long-term Outcomes of Injection Drug-related Infective Endocarditis Among People Who Inject Drugs. *J Addict Med*. 2020;14(4):282-286. doi:10.1097/adm.0000000000000572
41. Rammohan I, Gaines T, Scheim A, Bayoumi A, Werb D. Overdose mortality incidence and supervised consumption services in Toronto, Canada: an ecological study and spatial analysis. *The Lancet Public Health*. 2024;9(2):e79-e87. doi:10.1016/s2468-2667(23)00300-6
42. Nassau T, Kolla G, Mason K, et al. Service utilization patterns and characteristics among clients of integrated supervised consumption sites in Toronto, Canada. *Harm Reduction Journal*. 2022;19(1)doi:10.1186/s12954-022-00610-y
43. Wright T, Hope V, Ciccarone D, Lewer D, Scott J, Harris M. Prevalence and severity of abscesses and cellulitis, and their associations with other health outcomes, in a community-based study of people who inject drugs in London, UK. *PLoS One*. 2020;15(7):e0235350. doi:10.1371/journal.pone.0235350

© 2025 Padmanabhan P et al. *JAMA Network Open*.
