# Supplementary material for: Health, Costs, and Injection-Related Infections at a Hypothetical Overdose Prevention Center
Source: JAMA Netw Open. 2026 Jan 28;9(1):e2555965. doi: 10.1001/jamanetworkopen.2025.55965 (PMC12853211; doi:10.1001/jamanetworkopen.2025.55965)
Supplement: Supplement 2. — Data Sharing Statement [file jamanetwopen-e2555965-s002.pdf]

Data Sharing Statement

Padmanabhan. Health, Costs, and Injection-Related Infections at a Hypothetical Overdose Prevention Center. *JAMA Netw Open*. Published January 28, 2026.
doi:10.1001/jamanetworkopen.2025.55965

Data
Data available: No
